# Supplementary material for: Connecting energetics to dynamics in particle growth by oriented attachment using real-time observations
Source: Nat Commun. 2020 Feb 25;11:1045. doi: 10.1038/s41467-020-14719-w (PMC7042275; doi:10.1038/s41467-020-14719-w)
Supplement: Supplementary file 1 — Supplementary Information [file 41467_2020_14719_MOESM1_ESM.pdf]

Connecting energetics to dynamics in particle growth by oriented attachment using real-time observations

Liu and Nakouzi et al.

Supplementary Material for

Connecting energetics to dynamics in particle growth by oriented attachment using real-time observations

Lili Liu[1#], Elias Nakouzi[1#], Maria L. Sushko[1], Gregory K. Schenter[1], Christopher J. Mundy[1,2], Jaehun Chun[1,3] and James J. De Yoreo[1, 4*]*

1 Physical Sciences Division, Pacific Northwest National Laboratory, Richland, WA 99352

2 Department of Chemical Engineering, University of Washington, Seattle, Washington, 98195, USA

3 Benjamin Levich Institute, CUNY City College of New York, New York, New York 10031, USA

4 Department of Materials Science and Engineering, University of Washington, Seattle, Washington, 98195, USA

*Corresponding author: Jaehun.chun@pnnl.gov and James.deyoreo@pnnl.gov

Supplementary Figures

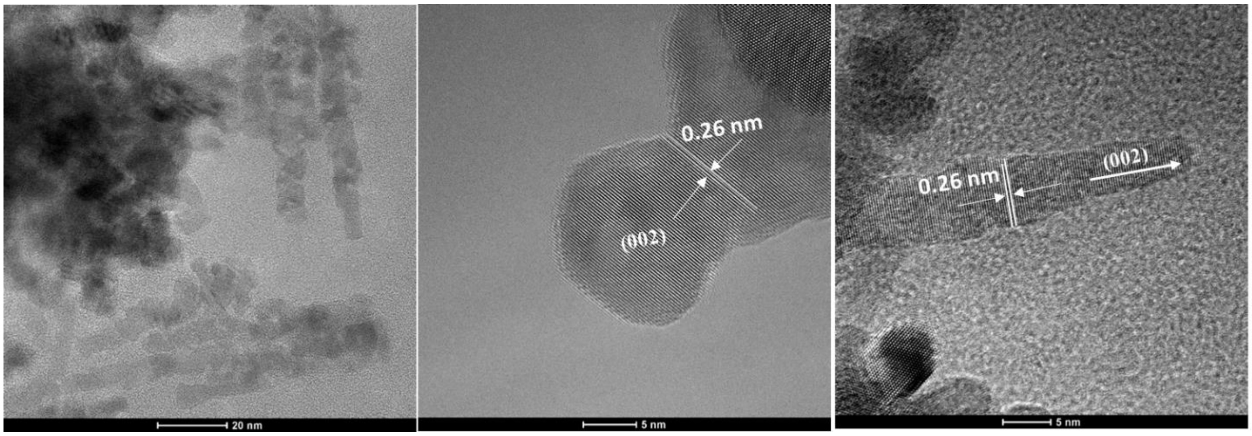

Supplementary Figure 1. TEM image of ZnO nanocrystals (synthesized 60°C for 2 h) kept at room temperature for three months in the presence of additional Zn^{2+} ions.

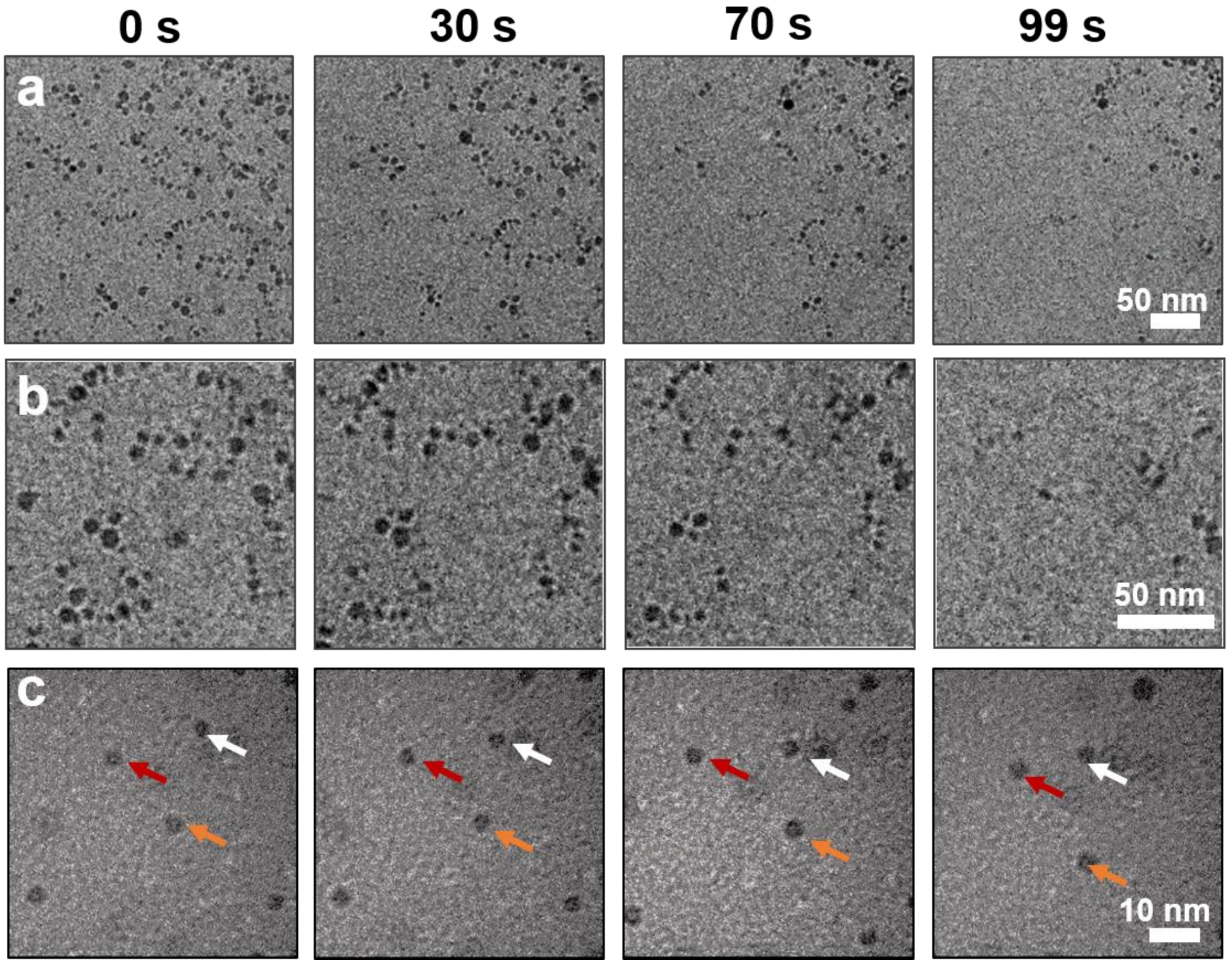

Supplementary Figure 2. TEM time-lapse images showing nanoparticle dissolution in (**a**) pure methanol and (**b**) $[Zn^{2+}]$ = 0.01 mM, as well as (**c**) stable particles in $[Zn^{2+}]$ = 1 mM

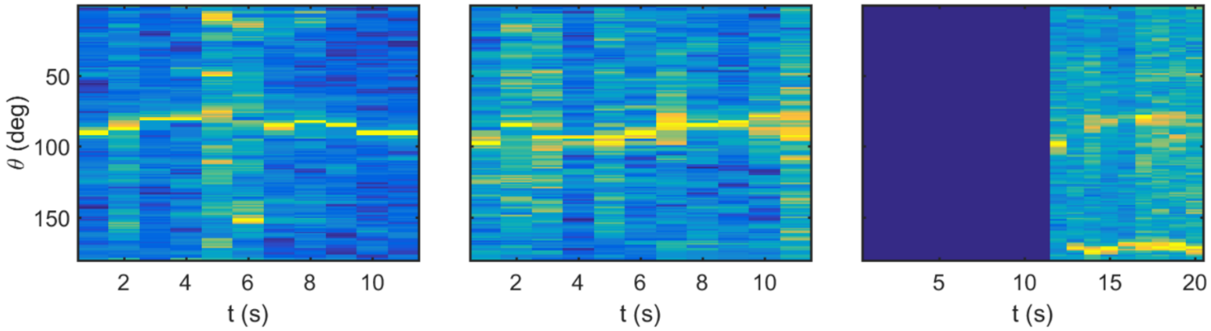

Supplementary Figure 3. Rotational dynamics during oriented attachment event of the two particles in Movie 3.

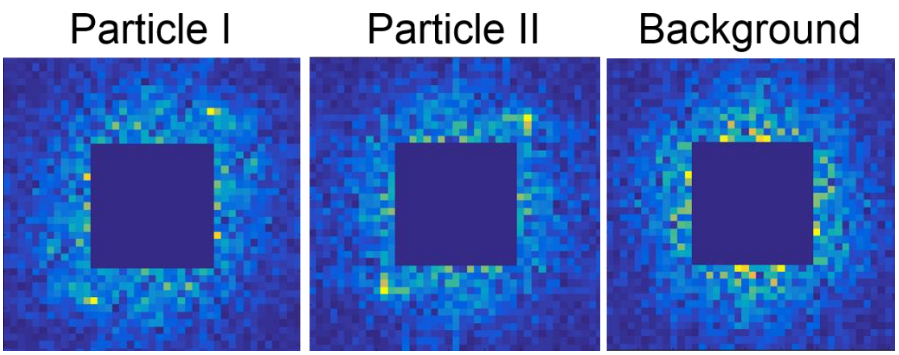

Supplementary Figure 4. Another example of oriented attachment, FFT spots at ~0.26 nm indicating ZnO crystal lattice show same orientation in two particles just prior to attachment.

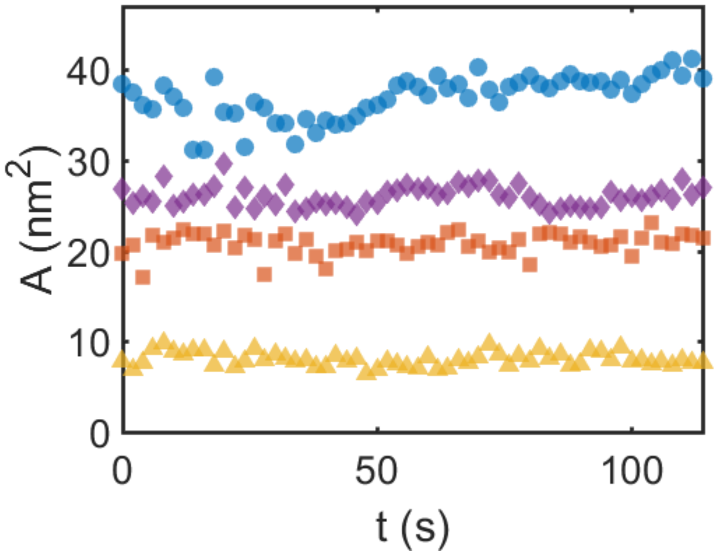

Supplementary Figure 5. Area versus time of ZnO nanoparticles. Data show that the individual particles do not grow or shrink during the course of the experiment, and only grow by particle attachment.

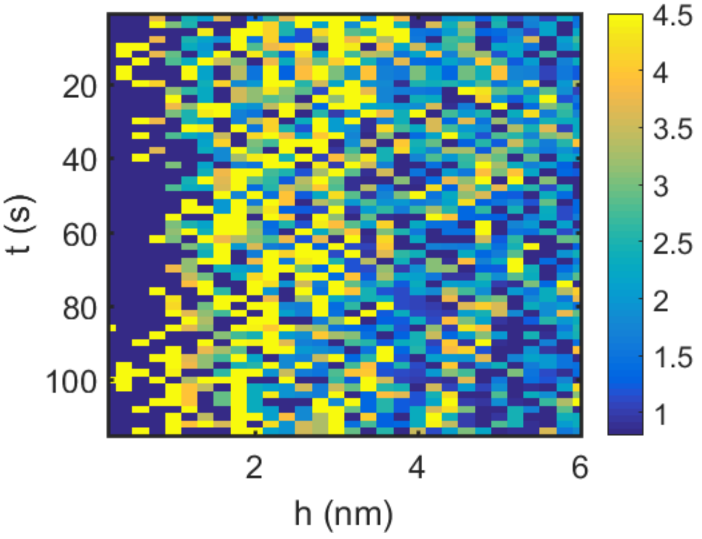

Supplementary Figure 6. Frame-by-frame evolution of *g(h)* during the course of the experiment. We do not observe a clear trend suggesting that the system is in a quasi-steady state condition.

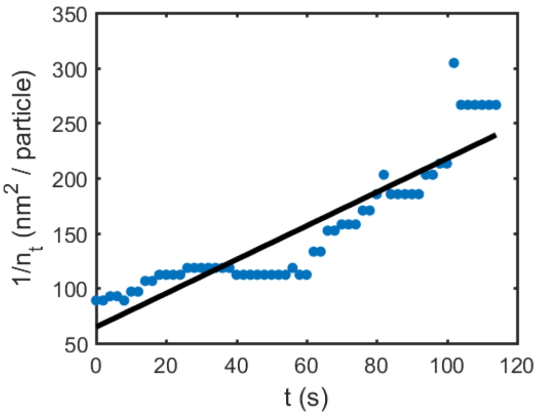

Supplementary Figure 7. Data representing monomer-monomer attachment events using second order kinetics. The plot allows us to calculate the rate constant k_{11}.

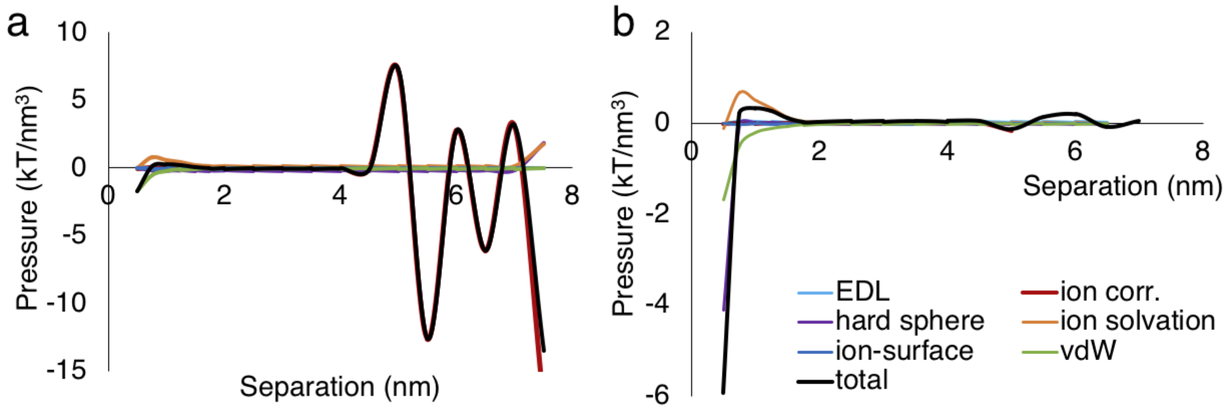

Supplementary Figure 8. Components of disjoining pressure between oppositely charged (0001)-(000-1) faces with (a) aligned pattern of charges and (b) with pattern of charges on one surface rotated by 20 degrees around the [001] axis in 1 mM zinc acetate dehydrate solution in methanol calculated using a full cDFT model. Curves represent electric double layer (light blue), ion correlation (red), ion solvation (orange), ion-surface (dark blue), hard sphere (purple), and van der Waals (green), and total (black) components of pressure.The EDL stands for electric double and vdW for van der Waals components of pressure, respectively. Ion solvation term stands for solvation pressure arising from ion-methanol interactions.

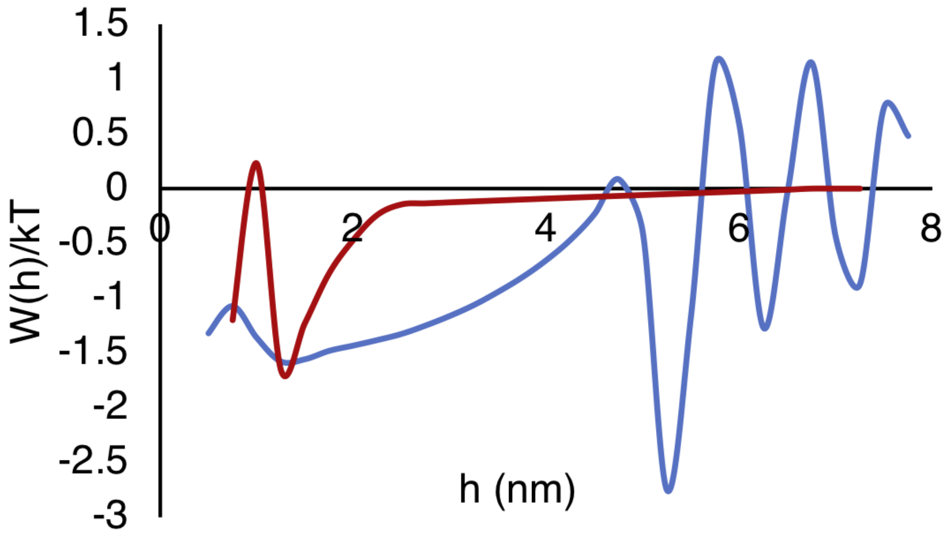

Supplementary Figure 9. Interaction potential between two spherical nanoparticles with radii 2 nm oriented along [001] direction with oppositely charged (i.e., (0001)-(000-1) faces, blue line) and like-charged (i.e., (0001)-(0001) faces, red line) surfaces.

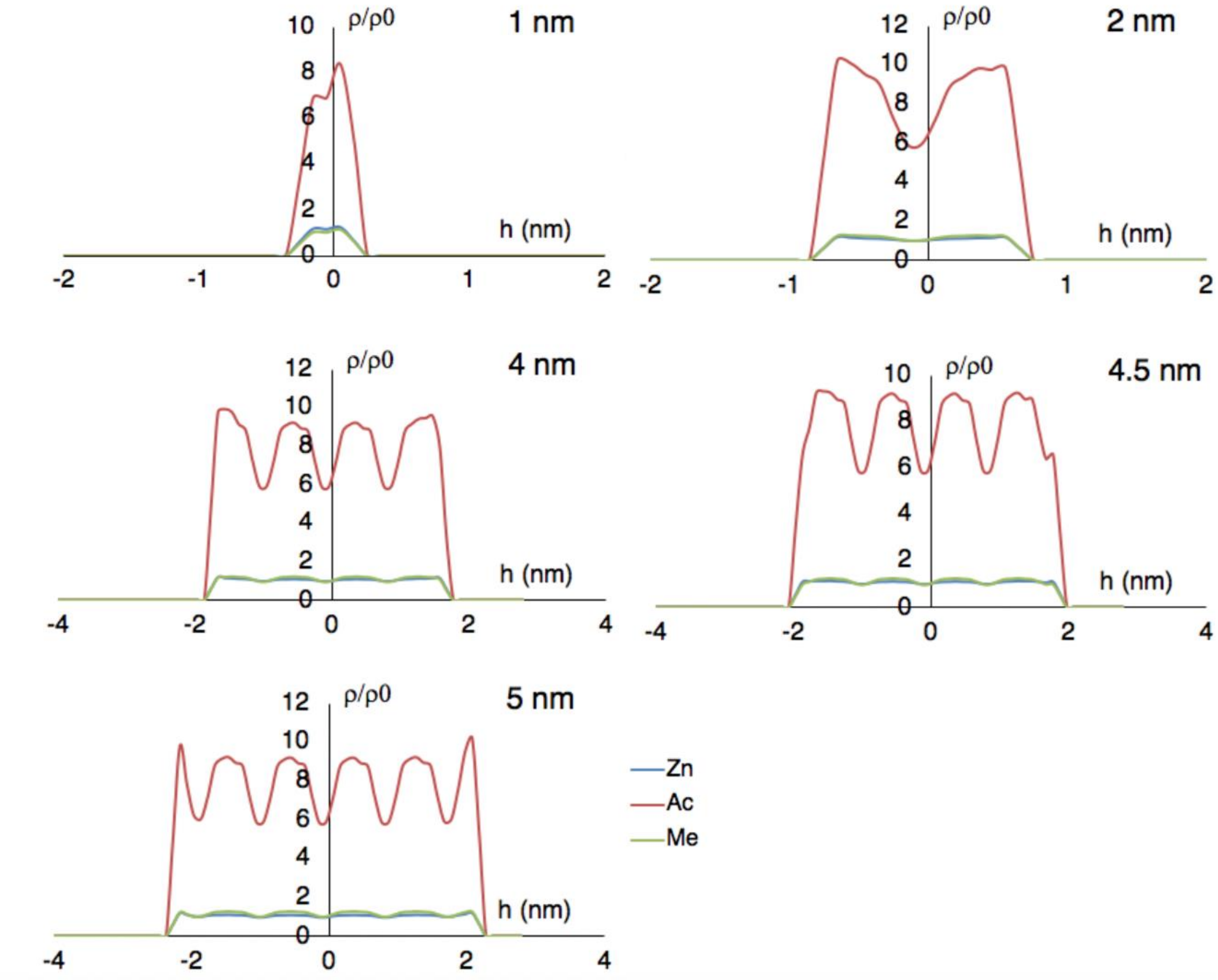

Supplementary Figure 10. Density profiles of ions and solvent confined between oppositely charged (0001)-(000-1) faces calculated using a full cDFT model for 1 mM zinc acetate dehydrate solution in methanol . The distances between the surfaces are shown in the insets.

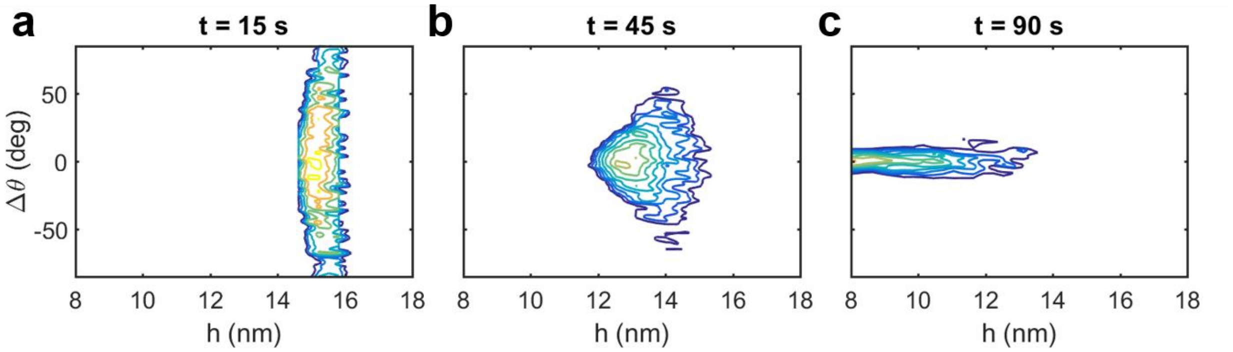

Supplementary Figure 11. Langevin dynamics simulation showing relative particle orientation and separation distributions. Snapshots at (a) t = 15 s, (b) 45 s, and (c) 90s.

Supplementary Discussion

To calculate the normalized particle area: $1/(A_1^{3/2}+A_2^{3/2})^{2/3}$

$$\text{We assume } V_F = V_1 + V_2$$

$$R_F^3 = R_1^3 + R_2^3$$

$$R_1 = R_2, \text{ then } R_F^3 = 2R_1^3$$

$$R_F^2 = (R_F^3)^{2/3} = 2^{2/3}\, R_1^2$$

$$\frac{A_F}{\frac{1}{2}(A_1 + A_2)} = \frac{2^{2/3} R_1^2}{\frac{1}{2}(R_1^2 + R_2^2)} = 2^{2/3}$$

$$A_F = \pi\, R_F^2 = \pi\,(R_1^3 + R_2^3)^{2/3}$$

$$R_1^3 = \left(\frac{\pi R_1^2}{\pi}\right)^{3/2} = \left(\frac{A_1}{\pi}\right)^{3/2}$$

$$A_F = \pi\left[\left(\frac{A_1}{\pi}\right)^{3/2} + \left(\frac{A_2}{\pi}\right)^{3/2}\right]^{2/3} = \left[A_1^{3/2} + A_2^{3/2}\right]^{2/3} \qquad (1)$$

We observe ZnO dissolution at in water and some methanol solutions under the influence of the electron beam. To rationalize the ZnO dissolution in methanol, we suggest the following hypothesis, previously reported by others[1-2]: The strong hydrogen bond between two CH_3OH molecules (mediated by one of the OH groups) may assist in breaking the methanolic C-O bond to produce CH_3O (methoxy), CH_3 (methyl), and H_2O according to $CH_3OH + CH_3OH \rightarrow CH_3O + CH_3 + H_2O$. Subsequently, electron beam irradiation on the resulting water can induce rapid formation of pH-lowering hydronium ions (H_3O^+) and solvated electrons (e_h^-) within the irradiated regions, which can be the driving force for ZnO dissolution.[3-4] This analysis provides a rationale for the faster rate of ZnO dissolution in water as compared to methanol and reinforces the conclusion that beam-induced chemical reactions cause major changes in the solution composition that are more important in LP-TEM dissolution studies than other effects, such as beam-induced temperature changes.[4]

We present here the pair conservation equation in quasi-2D, the ideal coagulation constant in 2D, and a scheme to estimate the numerical value. In our time-lapse images, the ZnO nanoparticles remain in the focal plane close to the TEM membrane, and hence do not diffuse into the bulk solution. Therefore, the equations for particle diffusion, collision frequency, and the corresponding coagulation rate (and thus an ideal coagulation constant) should be modified to reflect this quasi-2D nature.

In a typical three dimensional process,[5] the pair probability (p) becomes n^2 at $r\rightarrow\infty$, assuring a uniform particle density. Note that a number density (n) in the quasi-2D has a unit of [1/area] in contrast to [1/volume] for 3D. This boundary condition cannot be satisfied for 2D cases, conceptually analogous to a well-known Stokes paradox in 2D fluid mechanics problems.[6] We instead consider a finite circular boundary denoted as R (assuming the axi-symmetry as to a polar angle), which is a similar scheme used in the previous work by Saffman and Delbrück for Brownian motion in 2D;[7] that is, $p \rightarrow n^2$ at $r = R$. In principle, this finite boundary needs to satisfy $R >> a$ in order to ensure a uniform particle density at the boundary R, where a represents the particle size. The boundary condition is indeed equivalent to a notion that a time scale the particle diffuses over the boundary R is much larger than a typical coagulation time scale, which is indeed another representation of pseudo-steady condition of the particle density in the problem. This condition can be described by $(R/a)^2 >> 2/(a^2 n_0)$ where n_0 the initial particle concentration.

Starting from the pair probability conservation equation in 2D with the axi-symmetry as to a polar angle:

$$\frac{1}{r}\frac{\partial}{\partial r}\left(r\frac{\partial p}{\partial r}\right) = 0 \qquad (2)$$

which simply leads to $p = c_1 \ln r + c_2$ where c_1 and c_2 are integration constants. Applying the aforementioned boundary condition at $r = R$ and the condition of $p = 0$ at $r = 2a$ (i.e. particles do not exist at direct contact), we obtain:

$$c_1 = n^2\left[\ln\frac{R}{2a}\right]^{-1} \qquad (3)$$

and

$$c_2 = -n^2\left[\ln\frac{R}{2a}\right]^{-1}\cdot\ln 2a \qquad (4)$$

This leads to a pair probability profile given by:

$$p = \left[\frac{n^2}{\ln\left(\frac{R}{2a}\right)}\right]\ln\frac{r}{2a} \qquad (5)$$

Note that, using the experimental conditions (i.e., n_0 = 1.126×10^{-2} nm^{-2}, a = 2.2 nm), it was found that the condition $(R/a)^2 >> 2/(a^2 n_0)$ is satisfied with $R \geq 30$ nm; we used R = 30 nm throughout our analysis. Based on the pair probability, a diffusive flux at $r = 2a$ is described as:

$$2\pi r \left.\frac{\partial p}{\partial r}\right]_{r=2a} = \frac{2\pi n^2}{\ln\left(\frac{R}{2a}\right)} \qquad (6)$$

Similar to the original derivation by Smoluchowski for 3D case, a collision frequency between the pair of the particle that is a product of a relative particle diffusivity (i.e., two times of a relevant single particle diffusivity, $D_{||}$) and the diffusive flux is simply correlated to a kinetic equation of coagulation, based on a second order rate expression:

$$\text{collision frequency} = 2D_{||}\frac{2\pi n^2}{\ln\left(\frac{R}{2a}\right)} = -\frac{dn}{dt} = k_0 n^2 \qquad (7)$$

where k_0 is an ideal coagulation rate constant purely based on the diffusive motion of the particle in quasi-2D. Consequently, a new formulation for the 2D ideal coagulation rate constant can be represented by:

$$k_0 = \frac{2kT}{3\mu a\, G(a,h)}\frac{1}{\ln(R/2a)} \qquad (8)$$

To obtain the 2D ideal coagulation rate constant, we used an "effective" particle diffusivity considering two important physics: i) dominant tangential motions of the particle due to a much stronger hydrodynamic lubrication forces in the normal direction,[8] originated from the confined geometry associated with the underlying TEM membrane and ii) an effective viscosity near the interface (μ) different from a known nominal viscosity, $\tilde{\mu}$ (e.g., 10^{-3} Pa·sec for water). Note that such effective viscosity results from the anomalous diffusivities observed from our analysis on mean squared displacements (see below). The effective particle diffusivity ($D_{||}$) in the presence of the interface can be represented by:

$$D_{||} = \frac{kT}{6\pi\mu a\, G(a,h)} \qquad (9)$$

where the hydrodynamic resistivity of the particle in the presence of the interface, $G(a,h)$, is

$$G(a,h) = \left[1 - \frac{9}{16}\left(\frac{1}{1+\varepsilon}\right) + \frac{1}{8}\left(\frac{1}{1+\varepsilon}\right)^3 - \frac{45}{256}\left(\frac{1}{1+\varepsilon}\right)^4 - \frac{1}{16}\left(\frac{1}{1+\varepsilon}\right)^5\right]^{-1} \qquad (10)$$

Here, ε denotes a separation between the particle and interface (h), normalized by the particle radius.[8] A combination of Stokes-Einstein diffusivity (D_{SE}) and our direct observation for $D_{||}$

allows us to estimate the effective viscosity with recognizing a negligible contribution of $G(a,h)$ at $h/a \sim O(1)$ or smaller (i.e., $1/G(a,h) \sim O(1)$). That is,

$$\frac{D_{||}}{D_{SE}} = \frac{\frac{kT}{6\mu a\, G(a,h)}}{\frac{kT}{6\pi\tilde{\mu}a}} = \frac{\tilde{\mu}}{\mu G(a,h)} \approx \frac{\tilde{\mu}}{\mu} \qquad (11)$$

In our typical experimental condition, $\tilde{\mu}/\mu \sim 10^{-7}$ can be found from the above relation, which is qualitatively consistent with the previous studies. [9-10]

To estimate the coagulation rates, we use an approximation of the collision frequency. We assume a timescale for nanoparticle collision of ~45 s, which is reasonable based on the experimental data. Using the equation for collision frequency (*w*) derived above, we obtain $E/kT = -ln(r/w) \sim 3$, i.e. a relatively small barrier for nanoparticle attachment. Note that this result is robust to the selection of the collision timescale. For example, using a timescale of 6000 s – an overestimate of two orders of magnitude – only increases the barrier to ~7.7 *kT*.

In the following, we describe the calculation of timescales for rotational and translation motion of nanoparticles. The sufficient time for the rotational motion can be further elaborated by a scaling analysis. Since a decrease in the rotational mobility of the particle near the interface is much more pronounced in comparison to that due to a nearby particle,[8] a rotational time scale, τ_{rot}, can be estimated by $\tau_{rot} \sim \frac{1}{2D_r} = 4\pi\mu a^3 \left[\left(\frac{2}{5}\right)\ln\left(\frac{\tilde{h}}{a}\right) - 0.3817\right]/kT$,[8] where D_r is the rotational diffusivity and $\tilde{h}$ is a separation between the particle and interface. Here, the bracketed term denotes the inverse of the rotational mobility due to the presence of the interface. Utilizing the estimated effective viscosity near the interface and $\tilde{h}/a \leq 1$ as a reasonable approximation, τ_{rot} during the jump-to-contact (i.e., from *h*=2.2 to *h*=0 nm) is ~ 0.1 sec, whereas a time scale for the contact in the translational motion was ~ 60 sec from our observation (Figure 4A). This scaling clearly demonstrates the sufficient time to achieve a specific orientation for OA exists during the contact.

In the following, we describe the calculation of ZnO nanoparticle dipole moment. To estimate the dipole moment of ZnO nanoparticles, we fit the potential of mean force beyond 10 nm to $-2\mu^2/\varepsilon r^3$. For ε= 32 (corresponding to methanol), we find a dipole moment, μ, of 779 Debye by assuming aligned dipoles. We fit a straight line to the potential of mean force for $r < 13$nm, i.e., $W(r)$ =kT*(0.0795(r/nm)-1.45). Here $r=h+2a$ (or r is the magnitude of the r_{12} vector connecting the centers of dipole particle 1 and particle 2) where a=2.2 nm. As a consistency check, we consider the orientational dependence of the dipoles, constructing the potential, $V(r, \theta_1, \theta_2)$=kT*(0.0795 (r/nm)- 1.45) for $r < 13$ nm and $- \mu^2 *(3 \cos(\theta_1)*\cos(\theta_2)-\cos(\theta_1- \theta_2))/(\varepsilon r^3)$ for $r > 13$ nm. Here θ_1 and θ_2 are the orientation angles of the particle 1 and 2 with respect to along a line of centers vector, $\boldsymbol{r}_{12}$.

As a self-consistent check, we performed Langevin dynamics on the system:

$$\frac{d}{dt}r = -\frac{1}{\zeta_t}\partial_r W + \frac{1}{\zeta_t}f_t \qquad (12)$$

$$\frac{d}{dt}\theta_1 = -\frac{1}{\zeta_r}\partial_{\theta_1} W + \frac{1}{\zeta_r}\Gamma_1 \qquad (13)$$

$$\frac{d}{dt}\theta_2 = -\frac{1}{\zeta_r}\partial_{\theta_2} W + \frac{1}{\zeta_r}\Gamma_2 \qquad (14)$$

where f_t and Γ_i (i=1, 2) are zero mean Gaussian random force and torque respectively, satisfying:

$$\langle f_t(t) f_t(0)\rangle = 2\zeta_t k_B T \delta(t) \qquad (15)$$

$$\langle \Gamma_i(t) \Gamma_i(0)\rangle = 2\zeta_r k_B T \delta(t) \qquad (16)$$

Here, $1/\zeta_t$ and $1/\zeta_r$ are the translational and the rotational mobilities; in the hydrodynamic limit, ζ_t $=6\pi\mu a$ and ζ_r $=8\pi\mu a^3$. Note that we simply ignored hydrodynamic interactions associated with mobility for simplicity. Translational and rotational diffusion constants are respectively: D_t=kT/ζ_t and D_r=kT/ζ_r. For the simulations, we started at r=20 nm, and random orientations. We ended the simulations when $r < 1$ nm. We used the translational diffusion constant (D_t) as 2.99 nm^2/sec to be consistent with our experimental finding. We scaled appropriately the rotational diffusion constant: $D_r= D_t *(1.55\times10^7)/(9.98\times10^{-7})$.

In the following, we describe details of the classical density functional theory approach. Classical density functional theory (cDFT) was combined with Lifshitz theory of van der Waals forces and used to evaluate forces acting between two nanoparticles immersed in electrolyte solution. The total free energy functional has the form:

$$F(d) = F_0^{vdW}(d) + F_{hf}^{vdW}(d) + \sum F_i^{ion}(d) \qquad (17)$$

where the first two terms denote zero frequency (static) and high frequency (dynamic) van der Waals interactions between two ZnO nanoparticles immersed in electrolyte solution and the last term describes all ion-mediated mesoscopic and microscopic interactions described within classical density functional theory (see below) and *d*. is the distance between nanoparticle surfaces.

To avoid double counting microscopic interactions, mean-field non-retarded Lifshitz theory is employed for particle/particle van der Waals terms.[11] In particular, the high frequency van der Waals term is evaluated as

$$F_{hf}^{vdW}(d) = \frac{kT}{8\pi d^2}\sum_{n=1}^{\infty}\int_{r_n}^{\infty} x log\left[\left(1-\widetilde{\Delta}^2 e^{-x}\right)\left(1-\Delta^2 e^{-x}\right)\right]dx \qquad (18)$$

where $r_n = 2d\varepsilon_w^{1/2}\xi_n/c$ is a dimensionless factor quantifying the retardation screening and $\xi_n = 2\pi nkT/\hbar$ defines the eigenfrequencies at which dielectric function is evaluated, *c* is the velocity of light in vacuum and functions $\widetilde{\Delta}$ and Δ are defined as

$$\widetilde{\Delta} = \frac{s_w\varepsilon_a(i\xi_n) - s_a\varepsilon_w(i\xi_n)}{s_w\varepsilon_a(i\xi_n) + s_a\varepsilon_w(i\xi_n)} \qquad (19)$$

$$\Delta = \frac{s_w - s_a}{s_w + s_a} \qquad (20)$$

$$s_a = \sqrt{\left(\frac{x}{r_n}\right)^2 - 1 - \left(\frac{\varepsilon_a(i\xi_n)}{\varepsilon_w(i\xi_n)}\right)}, \quad s_w = \frac{x}{r_n} \qquad (21)$$

Note that since relative magnetic permeability of methanol and ZnO is close to 1,[12] we ignore the magnetic contribution in the above equation.

In the non-retarded limit $\Delta = 0$ and

$$\widetilde{\Delta} = \frac{\varepsilon_a(i\xi_n) - \varepsilon_w(i\xi_n)}{\varepsilon_a(i\xi_n) + \varepsilon_w(i\xi_n)} \qquad (22)$$

and the high frequency van der Waals free energy is given by

$$F_{hf}^{vdW}(d) = -\frac{kT}{8\pi d^2}\sum_{n=1}^{\infty}\sum_{m=1}^{\infty}\frac{\widetilde{\Delta}^{2m}(i\xi_n)}{m^3} \qquad (23)$$

The zero frequency contribution includes double-layer screening of zero frequency fluctuations and is calculated as

$$F_0^{vdW}(d) = \frac{kT}{4\pi}\int_{\kappa}^{\infty}\beta\, log\left(1 - \Delta^2 e^{-2\beta d}\right)d\beta \qquad (24)$$

$$\Delta \equiv \frac{\left(\sqrt{1-\left(\frac{\kappa}{\beta}\right)^2}\right)\varepsilon_a - \varepsilon_w}{\left(\sqrt{1-\left(\frac{\kappa}{\beta}\right)^2}\right)\varepsilon_a + \varepsilon_w} \qquad (25)$$

where κ is the Debye screening length in electrolyte solution and $\kappa \leq \beta < \infty$.

Here we described further details regarding the Classical Density Functional Theory approach. Methanol salt solutions are modeled as a dielectric medium with ε_m = 32.7 with certain densities of positively and negatively charged, spherical particles, representing the ions and neutral spherical particles representing methanol molecules. The density of spherical "methanol molecules" was 35 M, which gives an experimental methanol density. We used experimental crystalline ionic diameters for mobile ions: $\sigma_{Zn}{}^{2+}$ = 0.15 nm, $\sigma_{Ac}{}^{-}$ = 0.522 nm, and $\sigma_{methanol}$ = 0.285 nm.[3] The ion

charges were $q_{Zn}^{2+} = +2$, $q_{Ac}^{-} = -1$, and $q_{methanol} = 0$. All simulations were performed at 298 K temperature.

To determine the equilibrium methanol and ion distributions via cDFT and ion-mediated forces between nanoparticles, the total Helmholtz free energy functional is minimized with respect to the densities of all the species in the presence of rigid nanoparticles. For this optimization, it is convenient to partition the total free energy of the system into so-called ideal (F_{id}) and excess components (F_{ex}).[13] The ideal free energy corresponds to the non-interacting system and is determined by the configurational entropy contributions from methanol and small ions,

$$F^{id} = kT \sum_{i}^{N} \int_{\Omega} \left(\rho_i(\boldsymbol{r}) log \rho_i(\boldsymbol{r}) - \rho_i(\boldsymbol{r})\right) d\boldsymbol{r} \qquad (26)$$

where k is Boltzmann's constant, T is the temperature, ρ_i is the density profile of ion and methanol species i, N is the number of species, $\boldsymbol{r} \in \Omega$ is the ion coordinate, and Ω is the calculation domain. The excess free energy is generally not known exactly but can be approximated by

$F^{ex} = F^{ex}_{EDL} + F^{ex}_{hs} + F^{ex}_{el} + F^{ex}_{solv} + F^{ex}_{ion\_vdW}$

where $F^{ex}_{EDL} = F^{ex}_{C} + F^{ex}_{im}$ is describes first-order electrostatics and includes direct Coulomb term and image terms, F^{ex}_{hs} is the hard sphere repulsion term, F^{ex}_{el} is the electrostatic ion correlation term, F^{ex}_{solv} is the ion solvation term, and $F^{ex}_{ion\_vdW}$ describes ion-surface van der Waals interactions.

Disjoining pressure was calculated by varying separation between the particles with the step of 0.25 nm for separations lower than 3 nm and the step of 0.5 nm for larger separations. Then the resulting free energy per unit area was differentiated with respect to interparticle separation.

Calculations to implement the appreciable curvature of the particle were performed based on the surface element integration scheme18 which provides a higher accuracy than Derjaguin approximation. In this method interactions between surface elements facing each other are calculated and integrated over the equidistant shells over the surfaces facing each other, surfaces facing in the opposite directions and the corresponding cross terms are evaluated based on the separation dependence of free energy and potential between flat surfaces.

1. Poisson equation for first-order electrostatics. The electric double layer contribution to the free energy (F^{ex}_{EDL}) includes direct Coulomb and image interactions and is evaluated through the solution of Poisson's equation

$$-\nabla \cdot \varepsilon(\boldsymbol{r})\nabla\varphi(\boldsymbol{r}) = \rho_f(\boldsymbol{r}) + \sum_i q_i \, \rho_i(\boldsymbol{r}) \qquad (27)$$

for the electrostatic potential, $\varphi(\boldsymbol{r})$, where $\rho_f(\boldsymbol{r})$ is the fixed charge density on nanoparticle facets, $\rho_i(\boldsymbol{r})$ is the density of mobile species with charge q_i, $\varepsilon(\boldsymbol{r})$ is the dielectric coefficient equal to 32.7 in solution and 8.5 in the nanoparticles. The discrete distribution of charges on ZnO facets was constructed on a 2D grid using trilinear interpolation.

The EDL contribution to the free energy is then calculated as

$$F_{EDL}^{ex} = \sum_i \int_\Omega q_i \rho_i(\boldsymbol{r})\varphi(\boldsymbol{r})d\boldsymbol{r}$$

<u>2. Fundamental Measure Theory of excluded volume effects.</u> Hard sphere repulsive interactions describes ion and methanol many-body interactions in condensed phase due to density fluctuations. These interactions were described using a Fundamental Measure theory (FMT)[14]. The approach is based on the solution of the Ornstein-Zernike equation for direct correlation function using the Percus–Yevick approximation and yields the following form of the corresponding component of the free energy[15]:

$$F_{hs}^{ex} = kT\int \Phi_{hs}\,[n_\omega(\boldsymbol{r})]d\boldsymbol{r}$$

where the hard-sphere free energy density Φ_{hs} is a functional of four scalar and two vector weighted densities ($n_\omega(\boldsymbol{r})$) and has the form

$$\Phi_{hs}(r) = -n_0 \ln(1-n_3) + \frac{n_1 n_2}{1-n_3} + \left[\frac{1}{36\pi n_3^2} ln(1-n_3) + \frac{1}{36\pi n_3(1-n_3)^2}\right] n_2^3$$

$$-\frac{\boldsymbol{n_1}\cdot\boldsymbol{n_2}}{1-n_3} - \left[\frac{1}{12\pi n_3^2} ln(1-n_3) + \frac{1}{12\pi n_3(1-n_3)^2}\right] n_2(\boldsymbol{n_2}\cdot\boldsymbol{n_2}) \qquad (28)$$

where scalar (α = 0, 1, 2, 3) and vector (β = 1, 2) weighted densities are defined as

$$n_\alpha(\boldsymbol{r}) = \sum_i \int_\Omega \rho_i(\boldsymbol{r}')\omega_i^{(\alpha)}(\boldsymbol{r}'-\boldsymbol{r})d\boldsymbol{r}' \qquad (29)$$

$$\boldsymbol{n}_\beta(\boldsymbol{r}) = \sum_i \int_\Omega \rho_i(\boldsymbol{r}')\boldsymbol{\omega}_i^{(\beta)}(\boldsymbol{r}'-\boldsymbol{r})d\boldsymbol{r}' \qquad (30)$$

The “weight functions” $\omega^{(\alpha)}$ and $\omega^{(\beta)}$ characterizing the geometry of particles (hard sphere with

radius R_i for ion species i) are given by:

$$\omega_i^{(3)}(\boldsymbol{r}) = \theta(|\boldsymbol{r}| - R_i) \qquad (31)$$

$$\omega_i^{(2)}(\boldsymbol{r}) = |\nabla\theta(|\boldsymbol{r}| - R_i)| = \delta(|\boldsymbol{r}| - R_i) \qquad (32)$$

$$\boldsymbol{\omega}_i^{(2)}(\boldsymbol{r}) = \nabla\theta(|\boldsymbol{r}| - R_i) = \frac{\boldsymbol{r}}{r}\delta(|\boldsymbol{r}| - R_i) \qquad (33)$$

$$\omega_i^{(0)}(\boldsymbol{r}) = \omega_i^{(2)}(\boldsymbol{r})(4\pi R_i^2) \qquad (34)$$

$$\omega_i^{(1)}(\boldsymbol{r}) = \omega_i^{(2)}(\boldsymbol{r})(4\pi R_i) \qquad (35)$$

$$\boldsymbol{\omega}_i^{(1)}(\boldsymbol{r}) = \boldsymbol{\omega}_i^{(2)}(\boldsymbol{r})(4\pi R_i) \qquad (36)$$

In the preceding formulae (70), θ is the Heaviside step function with $\theta(x) = 0$ for $x > 0$ and $\theta(x) = 1$ for $x \leq 0$, and δ denotes the Dirac delta function.

3. Mean Spherical Approximation of ion-ion electrostatic correlations. To treat correlations resulting from electrostatic interactions between charged species on the same footing as those resulting from hard sphere excluded volume interactions Mean Spherical Approximation[16-17] was employed to solve the Ornstein-Zernike equation with respect to electrostatic direct correlation function. Taylor expansion of electrostatic free energy was cut after the second order. Then the electrostatic correlation component of the free energy (F_{el}^{ex}) is

$$F_{el}^{ex} = F_{el}^{ex}[\{\rho_i^{bulk}\}] - kT\int d\boldsymbol{r} \sum_{i=+,-} \Delta C_i^{(1)el}\left(\rho_i(\boldsymbol{r}) - \rho_i^{bulk}\right) -$$

$$\frac{kT}{2}\iint d\boldsymbol{r}d\boldsymbol{r}' \sum_{i,j=+,-} \Delta C_{ij}^{(2)el}(|\boldsymbol{r} - \boldsymbol{r}'|)\left(\rho_i(\boldsymbol{r}) - \rho_i^{bulk}\right)\left(\rho_j(\boldsymbol{r}') - \rho_j^{bulk}\right) \qquad (37)$$

where ρ_i^{bulk} are the bulk densities of charged species and the first and second-order direct correlation functions are defined as

$$\Delta C_i^{(1)el} = -\mu_i^{el}/kT \qquad (38)$$

$$\Delta C_{ij}^{(2)el}(|\boldsymbol{r}-\boldsymbol{r}'|) = \begin{cases} -\dfrac{q_i q_j e^2}{kT\varepsilon}\left[\dfrac{2B}{\sigma_{ij}} - \left(\dfrac{2B}{\sigma_{ij}}\right)^2 (|\boldsymbol{r}-\boldsymbol{r}'|) - \dfrac{1}{(|\boldsymbol{r}-\boldsymbol{r}'|)}\right], & (|\boldsymbol{r}-\boldsymbol{r}'|) \le \sigma_{ij} \\ 0, & (|\boldsymbol{r}-\boldsymbol{r}'|) > \sigma_{ij} \end{cases} \quad (39)$$

with

$$B = \frac{\left[\xi + 1 - (1+2\xi)^{\frac{1}{2}}\right]}{\xi} \quad (40)$$

$$\xi^2 = \kappa^2 o_{ij}^2 = \left[\frac{e^2}{\varepsilon kT}\sum_i q_i^2 \rho_i^{bulk}\right] o_{ij}^2 \quad (41)$$

In the above equations, μ_α^{el} is the chemical potential of the mobile ions, κ is the inverse Debye length and the contact distance $\sigma_{ij} = (\sigma_i + \sigma_j)/2$.

4. Ion solvation interactions. The short-range attractive solvation interactions between ions (denoted as "ion") and methanol "molecules" (denoted as "m") in electrolyte solution are given by

$$F_{solv}^{ex} = \frac{1}{2}\int_\Omega \int_\Omega d\boldsymbol{r}d\boldsymbol{r}' \sum_{\alpha,\beta=ion,m} \rho_\alpha(\boldsymbol{r})\,\rho_\beta(\boldsymbol{r}')\Phi_{\alpha\beta}(|\boldsymbol{r}-\boldsymbol{r}'|) \quad (42)$$

where $\Phi_{\alpha\beta}(|\boldsymbol{r}-\boldsymbol{r}'|)$ is the square-well potential

$$\Phi_{\alpha\beta}(|\boldsymbol{r}-\boldsymbol{r}'|) = \begin{cases} \infty, & |\boldsymbol{r}-\boldsymbol{r}'| < \sigma_{\alpha\beta} \\ -\tau, & \sigma_{\alpha\beta} \le |\boldsymbol{r}-\boldsymbol{r}'| < 1.2\sigma_{\alpha\beta} \\ 0, & |\boldsymbol{r}-\boldsymbol{r}'| \ge 1.2\sigma_{\alpha\beta} \end{cases} \quad (43)$$

with $\sigma_{\alpha\beta}$ equal to the contact distance between species α and β and depth τ equal to the scaled solvation enthalpy of the ions: τ_{Ac^-}= 0.0033 eV, $\tau_{Zn^{2+}}$= 0.011 eV.

5. Ion-surface van der Waals interactions. Lifshitz theory was used to calculate ion-surface van der Waals interactions.[18-19] It links ion dynamic polarizability and dielectric functions of the surface and the solvent giving the following potential for these interactions

$$V_i^{ion\_vdW}(d) = -\frac{\hbar}{(4\pi)^2 d^3}\int_\Omega^\infty d\xi \frac{\alpha_i^*(i\xi)}{\varepsilon_w(i\xi)}\varepsilon_a(i\xi) \quad (44)$$

where $\alpha_i^*(i\xi)$ is the excess polarizability of ion in methanol solution determined using time dependent DFT. The corresponding component of the excess free energy for two particles separated by a distance D along the z-axis is calculated as

$$F_{ion\_vdW}^{ex} = \int_{-D/2}^{0} dz \sum_i \rho_i(z)\, V_i^{ion\_vdW}\left(|z - \frac{D}{2}|\right) - \int_{0}^{\frac{D}{2}} dz \sum_i \rho_i(z)\, V_i^{ion_{vdW}}\left(z + \frac{D}{2}\right) \quad (45)$$

6. Density profiles. Density profiles are calculated within cDFT via the minimization of the total free energy functional F^{ex} with respect to the densities of all the species. The densities satisfy the following equation

$$\rho_i(\boldsymbol{r}) = \rho_i^{bulk}(\boldsymbol{r}) exp\left(-\frac{q_i\varphi(\boldsymbol{r})}{kT} - \frac{1}{kT}\frac{\delta\left(F_{el}^{ex} + F_{hs}^{ex} + F_{solv}^{ex} + F_{ion_{vdW}}^{ex}\right)}{\delta\rho_i(\boldsymbol{r})}\right) \quad (46)$$

where ρ_i^{bulk} is the bulk value for the atomic density of matrix and minor elements in alloy and oxygen atomic density in oxide, respectively. We solve Poisson's equation for the electrostatic potential ($\varphi(\boldsymbol{r})$). The resulting system of equations was solved iteratively to self-consistency using the numerical procedure described by Meng.[11] In particular, equilibrium ion density distributions were obtained using a relaxed Gummel iterative procedure. Convergence was considered to be achieved when the maximum difference between the input and the output density profiles between iterations was smaller than 10^{-6}.

We consider the meaning and implications of the oscillations seen in the full cDFT simulations for separations between 4 and 8 nm. These features are an unexpected prediction that have not been previously reported, nor are they obviously reflected in the experimental observations, though the small magnitude of the predicted barriers (~ kT for 2 nm particles) and the significant fluctuations in the experimentally derived RDFs may preclude such comparisons in the current data sets. Nonetheless, some discussion of their source and potential implications are in order. First, their absence in the primitive models is not surprising. In contrast to the full cDFT model used in this study, primitive cDFT models that do not treat solvent or ion-solvent interactions explicitly.

Second, the common qualitative feature of full and primitive cDFT models is the dominance of ion correlation forces in interparticle interactions at long- to medium-range separations. However, the differences in ion correlation forces calculated using these two cDFT models further emphasizes the importance of the role of solvent in producing a predictive model

of Zn-ion solvation interactions. The peaks and the wells in the disjoining pressure calculated in the full cDFT model correspond to the energetic cost and gain for accommodating a fraction or a whole number of structured solution layers between the surfaces, respectively (Supplementary Figure 10). This aspect of the model needs to be further validated with direct simulations (via Monte Carlo) of the full cDFT model. Moreover, the role of the discreteness of charge distribution on the interacting surfaces that will increase the ion correlation forces needs to be further explored in connection to orientation specificity.[1,2] Because these oscillations are directly related to the solution structuring predicted in the full cDFT model (see Supplementary Figure 10), our results suggest that such oscillations should be observed in experiments that probe solution-solid interfaces between two surfaces[2,3] in hopeful *qualitative* agreement with the full cDFT model. If they cannot be observed, then the possibility they are an artifact of the fully parameterized cDFT method itself needs to be explored.

7. System setup and Numerical implementation. cDFT equations are discretized by a finite difference scheme and solved iteratively using the Gummel method with relaxation. The algebraic multigrid method is applied to efficiently solve the Poisson equation. A novel strategy for calculating excess chemical potentials through fast Fourier transforms is implemented, which reduces computational complexity from O(N2) to O(NlogN), where N is the number of grid points. Integrals involving the Dirac delta function are evaluated directly by coordinate transformation, which yields more accurate results compared to applying numerical quadrature to an approximated delta function (see ref.25 for details).

The basic system geometry used for all cDFT simulations consists of two rectangular parallelepiped particles separated by a distance D along z-direction, the (x,y) area of the particles is 10 nm × 10 nm (Figure 5a in the main text). The particles are surrounded by the 10 nm3 solvent baths from both sides. Simulations were performed using constant particle number and Newman boundary conditions. The uniform distribution of all mobile species with their bulk densities in the available solution space of the simulation cell was used as the initial guess. The grid spacing of 0.075 nm, which corresponds to a fraction of particle diameters, was used.

Supplementary References:

1. Miller, A. V.; Kaichev, V. V.; Prosvirin, I. P.; Bukhtiyarov, V. I., Mechanistic study of methanol decomposition and oxidation on Pt(111). *J. Phys. Chem. C* **2013,** *117*, 8189-8197.
2. Chen, J.; Guo, Q.; Wu, J.; Yang, W.; Dai, D.; Chen, M.; Yang, X., Methanol decomposition on Co(0001): influence of the cobalt oxidation state on reactivity. *J. Phys. Chem. C* **2019,** *123*, 9139-9145.
3. Woehl, T. J.; Evans, J. E.; Arslan, I.; Ristenpart, W. D.; Browning, N. D., Direct in situ determination of the mechanisms controlling nanoparticle nucleation and growth. *ACS Nano* **2012,** *6*, 8599-8610.
4. Schneider, N. M.; Norton, M. M.; Mendel, B. J.; Grogan, J. M.; Ross, F. M.; Bau, H. H., Electron–water interactions and implications for liquid cell electron microscopy. *J. Phys. Chem. C* **2014,** *118*, 22373-22382.
5. Robins, M.; Fillery-Travis, A., Colloidal dispersions. *J. Chem. Technol. Biotechnol.* **1992,** *54*, 201-202.
6. Lamb, H., Hydrodynamics. *Dover Publications, New York* **1945,** *New York*.
7. Saffman, P. G.; Delbrück, M., Brownian motion in biological membranes. *Proc. Natl. Acad. Sci. U. S. A.* **1975,** *72*, 3111-3113.
8. Goldman, A. J.; Cox, R. G.; Brenner, H., Slow viscous motion of a sphere parallel to a plane wall—1 Motion through a quiescent fluid. *Chem. Eng. Sci.* **1967,** *22*, 637-651.
9. Li, T.-D.; Chiu, H.-C.; Ortiz-Young, D.; Riedo, E., Nanorheology by atomic force microscopy. *Rev. Sci. Instrum* **2014,** *85*, 123707.
10. Ortiz-Young, D.; Chiu, H.-C.; Kim, S.; Voïtchovsky, K.; Riedo, E., The interplay between apparent viscosity and wettability in nanoconfined water. *Nat. Commun.* **2013,** *4*, 2482.
11. Parsegian, V. A., Van der Waals Forces. *Cambridge University Press: New York, NY* **2006**.
12. Haynes, W. M., CRC handbook of chemistry and physics. **2014**.
13. Wu, J. Z.; Li, Z. D., Density-functional theory for complex fluids. *Annu.Rev.Phys.Chem* **2007,** *58*, 85-112.
14. Roth, R., Fundamental measure theory for hard-sphere mixtures: a review. *J. Phys. Condens. Matter.* **2010,** *22*, 063102.
15. Yu, Y. X.; Wu, J. Z., Structures of hard-sphere fluids from a modified fundamental-measure theory. *J. Chem. Phys.* **2002,** *117*, 10156-10164.
16. Blum, L., Mean spherical model for asymmetric electrolytes. *Mol. Phys.* **1975,** *30*, 1529-1535.
17. Hoye, J. S.; Blum, L., The mean spherical model for asymmetric electrolytes: thermodynamics and the pair correlation function. *Mol. Phys.* **1978,** *35*, 299-300.
18. Wernersson, E.; Kjellander, R., On the effect of image charges and ion-wall dispersion forces on electric double layer interactions. *J. Chem. Phys.* **2006,** *125*, 154702.
19. Ninham, B. W.; Yaminsky, V., Ion binding and ion specificity: the hofmeister effect and onsager and lifshitz theories. *Langmuir* **1997,** *13*, 2097-2108.
