# Supplementary material for: Connecting energetics to dynamics in particle growth by oriented attachment using real-time observations
Source: Nat Commun. 2020 Feb 25;11:1045. doi: 10.1038/s41467-020-14719-w (PMC7042275; doi:10.1038/s41467-020-14719-w)
Supplement: Supplementary file 3 — Description of Additional Supplementary Files [file 41467_2020_14719_MOESM3_ESM.pdf]

Description of Additional Supplementary Files

File Name: Supplementary Movie 1
Description: Zinc oxide particles attach to form elongated nanorods.

File Name: Supplementary Movie 2
Description: First example of oriented attachment of two zinc oxide nanoparticles.

File Name: Supplementary Movie 3
Description: Second example of oriented attachment of two zinc oxide nanoparticles.

File Name: Supplementary Movie 4
Description: Ensemble behavior of zinc oxide particle attachment.

File Name: Supplementary Movie 5
Description: Calculated particle trajectories from Langevin simulation of dipoledipole interactions.
